# Effect of prenatal EPA and DHA on maternal and cord blood insulin sensitivity: a secondary analysis of the mothers, omega 3, and mental health study

**DOI:** 10.1186/s12884-019-2599-6

**Published:** 2019-11-29

**Authors:** Joey A. England, Joses Jain, Bradley D. Holbrook, Ronald Schrader, Clifford Qualls, Ellen Mozurkewich

**Affiliations:** 1 Department of Obstetrics, Gynecology and Reproductive Sciences, McGovern Medical School, University of Texas Health Science Center at Houston, 6431 Fannin, MSB 3.286, Houston, 77030 USA; 2 Department of Obstetrics and Gynecology, University of New Mexico, 915 Camino de Salud NE, MSC10-5580, Albuquerque, 87131 USA; 3 Clinical and Translational Center, University of New Mexico, 915 Camino de Salud NE, MSC08-4635, Albuquerque, NM 87131 USA; 4 Department of Obstetrics and Gynecology, University of Michigan, L4001 Women’s Hospital, 1500 East Medical Center Drive, Ann Arbor, MI 48109-0276 USA

**Keywords:** EPA, DHA, Insulin sensitivity, Omega-3 fatty acids

## Abstract

**Background:**

We sought to determine whether prenatal supplementation with the omega-3 fatty acids eicosapentaenoic acid (EPA) or docosahexaenoic acid (DHA) would increase markers of insulin sensitivity in maternal or cord blood compared with placebo supplementation. A secondary aim was to evaluate the association of serum EPA and DHA fractions with adiponectin, leptin and the adiponectin:leptin ratio (ALR). We hypothesized that omega-3 fatty acid supplementation would increase markers of insulin sensitivity in maternal and umbilical cord plasma.

**Methods:**

We analyzed stored plasma samples collected from a prior 3-arm prospective, double-blinded, randomized controlled trial in which 126 women with singleton pregnancies between 12- and 20-weeks’ gestation were randomized to receive: 1) an EPA-rich fish oil supplement, 2) a DHA-rich fish oil supplement, or 3) a soy oil placebo. Maternal venous blood samples were collected at 12–20 weeks gestation (before supplementation) and at 34–36 weeks gestation. At delivery, cord blood was collected. Samples were analyzed using sandwich enzyme-linked immunosorbent assay kits to quantify leptin and adiponectin levels which were utilized to calculate the ALR, a proxy measure for insulin sensitivity.

**Results:**

We found no difference in adiponectin, leptin, and the ALR between the treatment and placebo groups at baseline, after supplementation, or in umbilical cord blood. In regression analyses, higher maternal serum DHA fraction was associated with increased ALR before (*p* = 0.01) and after (*p* = 0.04) DHA supplementation. There was no association of EPA fraction with any measure of insulin sensitivity. Cord blood DHA fraction was significantly associated with cord plasma leptin (*p* = 0.02).

Early pregnancy BMI was significantly associated with maternal leptin levels at baseline and in late pregnancy (*p* < 0.001) and was inversely associated with the ALR (p < 0.001). The ALR decreased significantly between the early and late pregnancy visits (p < 0.001). Pregnancy weight gain was inversely associated with the ALR (P. < 0.02).

**Conclusions:**

EPA- and DHA- rich fish oil supplementation had no effect on plasma markers of insulin sensitivity. However, maternal serum DHA fraction was significantly associated with markers of insulin sensitivity.

**Trial registration:**

https://clinicaltrials.gov/, registration number NCT00711971, 7/7/2008.

## Introduction

Insulin resistance is associated with several pregnancy-associated morbidities including gestational diabetes, increased birth weight, and increased risk of hypertensive disease [1]. Recent estimated prevalence of gestational diabetes mellitus was estimated as 6% among women who had a live birth in the United States between 2012 and 2016 [2]. Women with gestational diabetes have an increased risk of developing Type 2 diabetes in later life; a systematic review and meta-analysis involving 20 studies that included 675,455 women found that 12.5% of women with gestational diabetes developed Type 2 diabetes between 6 weeks after delivery and the end of the various follow-up periods [3]. Risks to the neonate include: hypoglycemia, respiratory distress, cardiac dysfunction and birth injury due to macrosomia [4]. Effects of maternal insulin resistance are not limited to the neonatal timeframe, but may also predispose the child to obesity and Type 2 diabetes [5].

It is not known whether dietary factors during pregnancy may influence the risk for patients to develop insulin resistance and gestational diabetes mellitus. In rodent models, dietary supplementation with omega-3 fatty acid docosahexaenoic acid (DHA) during pregnancy has been shown to be insulin-sensitizing [6]. In several studies in rodents, the omega-3 fatty acids DHA and eicosapentaenoic acid (EPA), have been shown to facilitate formation of insulin sensitizing adipokines such as adiponectin and reduce adipokines associated with insulin resistance [6, 7]. A recent meta-analysis of trials that evaluated the effect of omega-3 fatty acid supplementation on glucose control and insulin sensitivity among pregnant women with gestational diabetes mellitus found that supplementation resulted in reduced insulin resistance as measured by the HOMA-IR [8]. However, it is not known whether EPA- and DHA-rich fish oil supplementation would increase markers of insulin sensitivity in pregnant women without diabetes.

Other dietary factors may also influence insulin sensitivity. For example, Vitamin D supplementation has been reported to be insulin-sensitizing in non-pregnant individuals [9, 10]. Similarly, in a longitudinal study evaluating associations between vitamin D concentrations and insulin sensitivity during pregnancy, pregnant women who had blood concentrations of vitamin D that were deemed “sufficient” in early gestation had higher blood adiponectin concentrations than pregnant women deemed vitamin D insufficient [11].

The gold standard test to measure insulin resistance and beta-cell function is the Homeostatic Model Assessment (HOMA-IR test) [12]. This was first described in 1985 by Matthews. The approximating equation for insulin resistance used a fasting plasma sample and was delivered by the insulin-glucose product divided by a constant. This model correlates well with estimates using the euglycemic clamp method. However this test requires an overnight fast [13]. In situations in which the HOMA-IR test is impractical, the adipose secreted proteins, adiponectin and leptin, may be used as proxy measures of insulin sensitivity and resistance during pregnancy [14]. In particular, the adiponectin:leptin ratio (ALR) has been shown to be inversely related to the HOMA-IR test [14].

Our study objective was to determine whether maternal EPA- and DHA-rich fish oil supplementation would increase markers of insulin sensitivity in maternal and cord blood plasma compared with placebo supplementation. We hypothesized that maternal EPA- and DHA-rich fish oil supplementation would increase plasma adiponectin and increase the adiponectin:leptin ratio. We also sought to evaluate the association of serum DHA and EPA fraction as well as other potentially predictive variables available from the parent study, with adiponectin, leptin, and the ALR. We further hypothesized that maternal and umbilical cord DHA serum percentage would be associated with insulin sensitivity in maternal and fetal cord plasma. Our null hypothesis was that maternal fish oil supplementation would lead to no difference in plasma levels of adiponectin and leptin in either maternal or fetal samples compared with controls.

## Materials and methods

We conducted this analysis using maternal plasma samples collected from a prior investigation, “The Mothers, Omega-3, and Mental Health Study,” a 3-armed prospective, double-blinded, randomized controlled trial designed to test whether EPA- or DHA-fish oil supplementation would prevent perinatal depressive symptoms among women at risk. The full details of the trial have been previously described [15, 16]. In brief, participants with singleton pregnancies between 12- and 20-weeks gestation were recruited. Participants were selected based on an elevated risk for depression, defined as a past history of major depressive disorder, a history of postpartum depression, or an Edinburgh Postnatal Depression Scale (EPDS) score between 9 and 19. Exclusion criteria included age < 18, current major depressive disorder diagnosis, bipolar disorder or schizophrenia, substance abuse disorder, history of bleeding disorder, or clotting disorder requiring anticoagulation [15, 16]. Potential subjects taking antidepressant medications or omega-3 fatty acid supplements at baseline were excluded from participation in the study. However enrolled participants were allowed to initiate antidepressant medications during the course of the trial, if indicated [ 16].Women who met eligibility criteria and who consented to participate were randomized to one of three arms: 1) an EPA-rich fish oil supplement (1060 mg EPA plus 274 mg DHA), 2) a DHA-rich fish oil supplement (900 mg DHA plus 180 mg EPA), or 3) a soy oil placebo. Because the DHA- and EPA-rich fish oil capsules were not identical in appearance, all participants took some placebo capsules (double-dummy design). Full details of the randomization procedure and the supplements are described elsewhere [15, 16].

Maternal venous blood samples were collected after a 3 hour fast; baseline samples were collected at the time of study enrollment at 12–20 weeks gestation (visit 1), and again between 34- and 36-weeks’ gestation (visit 3). After delivery, a sample of fetal blood was collected from the umbilical cord (visit 4). Samples were centrifuged within 12 h of collection and plasma aliquots were stored at − 70 degrees Celsius.

Stored maternal and umbilical cord plasma samples from participants of the study were analyzed using commercially-available sandwich enzyme-linked immunosorbent assay kits (EMD Millipore, St. Charles, MO) to quantify leptin and adiponectin levels according to the manufacturer’s protocols [17, 18]. The manufacturer’s reported within- and between-assay coefficients of variation were 3.3 and 5.7% respectively for adiponectin and 2.6 and 3.75% respectively for leptin [17, 18]. The only variation from the manufacturer’s recommended protocol was a dilution of fetal adiponectin samples to 1:1000 as opposed to 1:500 for maternal samples, based on higher fetal levels of adiponectin. Absorbance of both adiponectin and leptin were measured at 450 nm and 690 nm and the results compared. 25-hydroxyvitamin D levels, as well as DHA and EPA fractions in maternal and cord blood serum had been previously assayed [16, 19].

ETHICS: The procedures followed in the parent study were approved and conducted in accordance with the institutional review boards of the University of Michigan Medical Center, Ann Arbor, MI, St. Joseph Mercy Hospital in Ypsilanti, MI, and The University of New Mexico Health Sciences Center Human Research Protections Office. All participants gave written informed consent to participate in the study. Participants granted written permission for blood sample analyses for lipids and for inflammatory markers. The trial was registered on 7/7/2008 at clinicaltrials.gov: NCT00711971 under the title: “Does Fish Oil Prevent Depression in Pregnancy and Postpartum”.

The analyses of de-identified blood samples and data described in this manuscript were approved as an amendment to the parent study ((HUM00004684) by the IRB at the University of Michigan Medical Center. The secondary blood sample analyses described in this manuscript were judged exempt by the University of New Mexico Health Sciences Center Human Research Protections Office (HRPO12–466).

STATISTICS: Demographic variables were compared among three randomized groups (EPA-rich fish oil, DHA-rich fish oil, and placebo) as means and standard deviations if continuous or ordinal scale using 1-way ANOVA and as frequencies using Fisher’s exact test. We computed medians, and interquartile ranges for each outcome parameter, adiponectin, leptin and the adiponectin:leptin ratio (ALR). Using raw (unadjusted) data, in order to test whether these parameters changed over time or were different between groups, we performed repeated measures ANOVA with group as grouping factor and visit as repeated factor. The analysis of the ALR was also performed using repeated measures ANOVA with time (enrollment before supplementation, and after supplementation) as a repeated factor and 3 groups as a grouping factor. This was an intent-to-treat analysis in that all available data were used. Outcomes adiponectin, leptin, and constituents of ALR, were analyzed similarly. Because box plot analysis of the data revealed non-normality, we also analyzed these data after square root transformation. In addition, each outcome after supplementation was analyzed among the 3 groups using linear regression methods (multivariable regression) with predictors being baseline measures of the given outcome and covariates (BMI at enrollment, maternal weight gain, age, and ethnicity).

As an alternative analysis we also computed the change in the maternal adiponectin, leptin, and the adiponectin:leptin ratio, and compared the change among the three treatment groups. One- way ANOVA were followed by Fisher’s post hoc t tests. We used ANCOVA to evaluate initiation of antidepressant medications during the trial as a predictor of the change in adiponectin, leptin, and the ALR.

We performed post-hoc analyses of the relationship of adiponectin, leptin, and the ALR with the following predictive variables: serum DHA fraction, serum EPA fraction, plasma fractions and 25-OH vitamin D concentrations, maternal BMI at study entry, maternal weight gain, and age at study enrollment were performed using multivariable linear regression. The analysis of the effect of maternal weight gain on the outcome measures was adjusted for maternal weight at study entry.

For these exploratory analyses, the three treatment groups and two maternal time points were pooled unless otherwise specified. We calculated the effect of a one unit increase in each of the predictive variables on the ALR using the two-tailed student’s t distribution.

We calculated the medians and interquartile ranges for adiponectin, leptin and the ALR in umbilical cord blood. We used raw unadjusted data, as well as square root transformation to evaluate difference according to group assignment using ANOVA. Covariate adjustments were done for birth weight and length of gestation. The association of DHA and EPA fractions, as well as 25-OH vitamin D concentrations in cord blood with measures of insulin sensitivity were analyzed by multivariable linear regression. *P*-values ≤0.05 were considered statistically significant.

SAMPLE SIZE CONSIDERATIONS: The current study is a secondary analysis of data from a randomized controlled trial for which the primary outcome was depressive symptoms as measured by the Beck Depression Inventory [15, 16]. The sample size was chosen based on a hypothesized difference in the BDI score. For this secondary analysis, we performed a post- hoc power analysis to determine the power of the current study to detect a difference in maternal and cord blood adiponectin concentrations based on adiponectin values available in the literature. From Luo et al. [20] we assumed that baseline adiponectin level would have a baseline standard deviation of approximately 3 micrograms/ml. Assuming a modest within subject correlation of 0.7, change scores from the enrollment (12–20 weeks) to post supplementation (34–36 weeks) should have a standard deviation of approximately 2.3 micrograms/ml. For this standard deviation the minimum detectable mean difference between 2 groups from a two-sample t-test with significance of 0.05 and power of 0.8 is 1.5 micrograms/ml where the sample sizes for the two groups both are 36.

For the comparisons of adiponectin, leptin, and the ALR in cord blood, we assumed an adiponectin standard deviation of approximately 8.0 micrograms/ml, based on Luo, et al. [20] For a two sample t test with this standard deviation a sample size of 32 per group gives significance level of 5% and power of 80%; the minimum detectable difference is 5.7 micrograms/ml. The proposed analysis should have superior power and a smaller detectable difference.

## Results

There were 126 participants who were randomized, of whom 118 completed the trial. For this secondary analysis, there were 113 plasma samples available at trial entry, 109 samples available post-supplementation (34–36 weeks) as well as 98 cord blood samples. The flow of participants and samples is shown in Fig. 1.

The baseline characteristics of the three randomized groups are shown in Table 1. There were no significant differences in the baseline characteristics between the three randomized groups.

**Table 1:** Demographics

Variable	EPA-rich fish oil group *N* = 36	DHA-rich fish oil group *N* = 37	Soy oil placebo group *N* = 41	Statistical Significance
Maternal age at enrollment	29.9+/−5.2	30.7+/−4.4	30.4+/− 5.9	NS*
White race	32	28	34	NS^‡^
BMI at enrollment	28.5+/−6.3	28.5+/−6.7	27.7+/−8.2	NS*
Gestational age at enrollment (weeks)	15.9/2.7	16.9/2.3	16.0/2.3	NS*
Gravidity	2.42/1.36	2.55/1.22	2.41/2.02	NS^‡^
Parity	0.87/0.84	1.08/0.94	0.85/1.20	NS^‡^

NS = not significant *1-way ANOVA ^‡^ Fisher’s Exact test.

Note: This table represents the subset of the total cohort with samples available for analysis. The total study demographics have been previously described [16]

Our primary intent-to-treat analysis using repeated-measures modeling showed no significant difference in adiponectin, leptin, and the ALR in maternal plasma between the treatment groups and placebo group at baseline or after supplementation. (Table 2). These results were unchanged after square root transformation of the data.

**Table 2:** Adipokine values before and after supplementation according to treatment group

Parameter	Visit	EPA	DHA	Placebo	RM ANOVA *P* values		
					Group	Visit	Interaction
Adiponectin ng/ml	1	24.15 (17.72,29.70)	26.15 (14.94,38.04)	25.29 (15.56,33.69)	0.83	< 0.001	0.97
	3	18.69 (13.79,22.05)	19.91 (12.95,27.90)	18.92 (14.20,25.71)			
Leptin ng/ml	1	27.93 (17.38, 35.77)	22.04 (11.46,34.44)	19.72 (15.04,28.34)	0.13	0.32	0.86
	3	28.70 (19.69, 47.33)	24.38 (15.97,33.34)	23.91 (16.97,32.72)			
A:L Ratio	1	0.83 (0.41,1.63)	1.44 (0.44,2.32)	1.17 (0.52,1.97)	0.60	< 0.001	0.92
	3	0.54 (0.34, 0.91)	0.77 (0.50,1.54)	0.75 (0.36,1.26)			

Note. Cell formats are median (IQR). Reported adiponectin concentrations after 1:500 dilution

Of interest, for the total cohort adiponectin significantly decreased between enrollment and 33–36 weeks gestation (p < 0.001) while leptin significantly increased during the same time period (*p* = 0.008). The ALR significantly decreased during this same time period (*p* < 0.001) indicating increasing insulin resistance with advancing pregnancy. The magnitude of decrease in the ALR was 31%.

Detailed comparison of change (delta) among the 3 treatment groups found no significant differences among the three groups of delta adiponectin, leptin, or the ALR. (Table 3).

**Table 3:** Delta Adiponectin, Leptin, and ALR according to treatment group

Parameter	EPA	DHA	Placebo	*P* values
				Group
Δ Adiponectin ng/ml	−5.35 (−30.0,6.43)	−5.17 (−19.92,16.44)	−6.66 (−41.10,15.27)	0.75
Δ Leptin ng/ml	2.83 (−27.93, 35.03)	−0.05 (−40.93,17.60)	3.39 (−25.19,19.48)	0.45
Δ A:L Ratio	−0.47 (−5.47,0.37)	− 0.51 (−4.16,0.40)	−0.68 (− 6.11,0.61)	0.68

Note: P-values for an among group difference were computed using 1-way ANOVA. Reported adiponectin concentrations reflect 1:500 dilution

Initiation of antidepressant medications during the trial was not significantly predictive of the ALR (*P* = 0.13).

We performed regression analyses to explore the relationships between measured serum DHA and EPA fraction at enrollment and after supplementation with measures of insulin sensitivity. Variables included in the model included BMI at enrollment, DHA fraction, EPA fraction, vitamin D concentration, and age at enrollment. After adjusting for BMI and maternal age, higher maternal serum DHA fraction was positively associated with increased ALR before (*p* = 0.01) and after (*p* = 0.04) supplementation. DHA fraction was positively associated with adiponectin after supplementation (*p* = 0.03); there was a non-significant trend towards association of DHA with adiponectin at study enrollment. There was no significant association of EPA with any measure of insulin sensitivity. Vitamin D concentration was also positively associated with adiponectin (*p* < 0.05) at enrollment but was not significantly associated with adiponectin after supplementation.

Early pregnancy BMI was positively associated with maternal leptin levels at baseline and in late pregnancy (*p* < 0.001) and was inversely associated with the ALR (p < 0.001). ALR decreased significantly between the early and late pregnancy visit (p < 0.001). After adjusting for baseline weight, maternal weight gain between the early pregnancy visit and the late pregnancy visit was positively associated with plasma leptin (*p* < 0.01) and negatively associated with the ALR (*p* < 0.02). .

The effect of a one unit increase in each of the variables of interest on the ALR is shown in Table 4.

**Table 4:** Variables predictive of the change (delta) in the ALR

Variable	Estimate	95% CI
Pregnancy Weight Gain (1 kg)	− 0.04 (*p* < 0.02)	[−0.07, − 0.007]
Study Enrollment BMI	− 0.03 (*p* < 0.0001)	[− 0.04, − 0.02]
After Supplementation BMI	−0.02 (*p* < 0.001)	[− 0.03, − 0.01]
Study Enrollment DHA (1 unit)	0.2 (p < 0.02)	[0.02, 0.17]
After Supplementation DHA (1 unit)	0.1 (p < 0.02)	[0.003, 0.112]

Note. *P*-values and Confidence Intervals (CI) were based on two-tailed student’s t distribution

There were no differences in adiponectin, leptin, or the ALR in umbilical cord blood according to maternal treatment allocation group. (Table 5) Regression analysis was performed to evaluate the association of EPA fraction, DHA fraction, vitamin D concentration, birth weight, gestational age at delivery, and mode of delivery with adiponectin, leptin and the ALR in umbilical cord blood. There were no significant associations between the variables of interest on cord blood adiponectin or the ALR. However, birth weight and cord blood DHA fraction were significantly associated with serum leptin (*p* = 0.02, both). The association of birth weight with cord blood leptin persisted when controlling for DHA fraction, and the association of DHA fraction with cord blood leptin persisted when controlling for birth weight.

**Table 5:** Cord blood adipokine values according to treatment group

Parameter	EPA	DHA	Placebo	Group
Adiponectin ng/ml	52.07 (38.99,70.98)	55.67 (38.81,68.34)	52.40 (43.42,65.70)	0.93
Leptin ng/ml	9.25 (4.60,13.55)	7.98 (4.26,19.84)	7.10 (3.23,13.71)	0.56
A:L Ratio	5.85 (3.63,11.19)	6.21 (2.47,15.07)	8.69 (4.03,19.36)	0.27

Note. Cell formats are median (IQR)

## Discussion

The main finding of our study was that DHA- and EPA- rich fish oil supplementation had no significant effect on plasma markers of insulin sensitivity, compared to placebo. However, maternal serum DHA fraction and plasma 25-OH vitamin D were significantly associated with markers of insulin sensitivity. Of interest, we found that maternal BMI and weight gain negatively affect insulin sensitivity as measured by the ALR, increased maternal leptin and decreased maternal adiponectin levels.

The findings of our study were similar to that of the Haghiac trial [21], which randomly assigned 49 overweight pregnant women to omega-3 fatty acid supplementation conferring 2 g DHA plus EPA daily; this study found no difference in adiponectin, leptin, or HOMA-IR between the randomized groups. Similarly, a pilot study of 3.36 g of omega-3 fatty acid supplementation versus matched placebo among 62 hypertensive male adults found no effect of supplementation on serum adiponectin [22].

Our findings contrast with those of the ComparED study [23], which randomized 154 otherwise healthy obese non-pregnant adults to receive 2.7 g of EPA or DHA versus corn oil placebo. In that population, the investigators found that DHA, but not EPA supplementation significantly increased serum adiponectin, compared with corn oil placebo [23].

Our exploratory analysis of variables predictive of markers for insulin sensitivity found maternal vitamin D concentrations to be associated with markers of insulin sensitivity, similar to the finding of Benaim, et al. [11]

Similar to our findings, in a Mexican cohort, Solis-Paredis, et al., found maternal leptin levels to be significantly associated with maternal pregnancy weight gain [24]. A birth cohort study by Logan, et al., also found maternal pregnancy weight gain to be associated with decreased insulin sensitivity [25].

Contrary to our expectation, we found serum DHA fraction to be significantly associated with higher cord blood leptin levels. In other investigations, cord blood leptin has been significantly associated with higher birth weight [26, 27]. Elevated cord blood leptin may confer risk for childhood obesity, through developmental programming [26].

Our study had several strengths and limitations. Strengths of our study included the randomized design as well as the double-blinded nature of the trial. Availability of both maternal blood and umbilical cord blood was also a strength, although our study design allowed for only one post-supplementation assessment of insulin sensitivity, timed at 34–36 weeks gestation. However, our study was limited in the respect that our subjects were selected based on predisposition to depression and may not be representative of the general population of pregnant women. Similarly the sample size for the study was determined based on the unrelated primary outcome of depressive symptoms.

## Conclusions

In conclusion, prenatal EPA- and DHA rich fish oil supplementation had no significant effect on markers of insulin sensitivity during pregnancy. However maternal DHA fraction and 25-OH vitamin D concentration were associated with greater insulin sensitivity in pregnancy. Maternal BMI and pregnancy weight gain were related to lower insulin sensitivity during pregnancy.

Future studies may include a well-powered randomized controlled trial of maternal DHA and/or vitamin D supplementation in women at risk for insulin resistance in order to augment insulin sensitivity.

## Abbreviations

ALR: Adiponection/leptin ratio
DHA: Docosahexaenoic acid
EPA: Eicosapentaenoic acid
